# The Use of Dynamic Cognitive Behavioural Therapy (DCBT) in Social Anxiety Disorder (SAD): A Theoretical Integration Initiative

**DOI:** 10.3390/medicina58121759

**Published:** 2022-11-30

**Authors:** M. Siyabend Kaya

**Affiliations:** 1 School of Psychology and Clinical Language Sciences, University of Reading, Reading RG6 6AL, UK; m.s.kaya@pgr.reading.ac.uk; 2 Department of Psychology, Abdullah Gül University, Kayseri 38080, Turkey

**Keywords:** social anxiety, dynamic cognitive-behavioural therapy, psychodynamic therapy, cognitive-behavioural therapy, integrative therapy

## Abstract

Psychotherapy theorists can often become fervent advocates of the schools they follow and place the doctrines of the theories they adopt above all else. This situation can sometimes turn into a war of theories between researchers as well. However, therapists should not aim to shape therapy sessions according to their methods but to use them in line with clients’ needs. Although it is emphasised that the integration of both psychoanalytic and cognitive behavioural therapy techniques, which is going to be named dynamic cognitive behavioural therapy (DCBT) in this case report, will provide more effective and permanent treatment, a discernible gap exists regarding the integration of these theories and their use in psychotherapy. Taking into account this gap, it is considered important to use this approach with a client who has a social anxiety disorder (SAD). Therefore, this study aims to describe the almost forgotten DCBT approach step by step through a case report and reveal the effectiveness of this approach. As a result, DCBT seems to be effective in the treatment of SAD.

## 1. Introduction

Social anxiety disorder (SAD) is one of the most common anxiety disorders that increases rapidly, and disproportionately affects young people [1]. After depression and alcohol dependency, it is the third most prevalent psychological disorder According to the epidemiological reports, its current prevalence is between 5 and 10 percent, and its lifetime prevalence is 8.4 to 15 percent [3]. However, the researchers have found that the global prevalence of social anxiety is significantly higher than previously reported, and more than one-third of respondents meet the threshold criteria for social anxiety disorder (SAD), based on their prevalence study from seven countries [1]. If left untreated, it can lead to many severe problems in people’s lives [4].

Psychotherapy, pharmacotherapy, and a combination of both are established treatment methods for anxiety disorders [5]. Among psychotherapy methods, the most used treatment methods are cognitive behavioural [6] and psychodynamic theories [7]. Cognitive-behavioural therapy is a type of psychotherapy that combines cognition and learning theories with treatment strategies drawn from cognitive therapy and behaviour therapy and aims to uncover and improve the client's maladaptive thought processes and harmful behaviours using cognitive restructuring and behavioural approaches [8]. Psychodynamic therapy is a form of psychotherapy that derives from the psychoanalytic tradition, emphasising the role of the unconscious and transference analysis in treatment and focusing on the change and development of the individual [9]. It is possible to come across numerous research reports stating that both cognitive-behavioural therapy (CBT) and psychodynamic therapy (PDT) are effective. For example, Lindegaard and his colleagues [10] recently investigated the effectiveness of the CBT and PDT protocols they prepared online for clients with SAD. The researchers stated that both treatment methods were promising, and clients showed similar improvements [10].

On the other hand, some studies state that both CBT [11] and PDT [12] do not work from time to time. However, when the studies conducted are examined, it is seen that psychodynamic therapy is criticised more heavily [13,14]. One journal even went as far as stating that psychoanalytic case reports should not be published because they have no scientific value [15]. However, PDT is a therapy method that has been proven to be as effective as many other theories, especially CBT [16]. In fact, many studies in the literature reveal PDT is even more effective than CBT from time to time [17]. However, biased approaches to PDT can prevent us from understanding its importance [18].

When looking at the literature, it is seen that researchers can perceive the differences between theories as a deficiency and even turn it into a battle of theories. For example, a comprehensive meta-analysis study concluded that CBT loses its effectiveness as time goes on [19]. Immediately afterwards, another group of researchers conducted a new meta-analysis study to prove that this was not the case. This time, researchers claimed that the results of the previous meta-analysis could be spurious, and concluded that CBT does not lose its efficacy [20]. As the saying goes, researchers are turning the issue into a clash of elephants, as emphasised in an African proverb [21], as this battle does nothing but crush the grass. The therapists’ aims should not be to sculpt clients according to their theories but to use them in line with their client’s needs. Integrative therapy methods are an evolving search to meet the needs of clients.

Integrative psychotherapy does not aim to unite all psychotherapy methods into a single theory but aims to develop a new field for a common goal by taking advantage of the power of different approaches [22]. It emphasises that one theory can close the weaknesses of another, and thus the psychotherapy process can become more beneficial for clients. For example, one of the biggest criticisms directed by researchers of CBT is that it does not give enough importance to therapeutic communication [23,24]. Moreover, it is stated that dynamically oriented therapies do not care about the conscious process as much as the unconscious process [25]. In this sense, DCBT, which is a type of integrative psychotherapy that combines CBT and PDT and emphasises the role of both unconscious and active mental processing in human functioning [26], is a candidate to be a synthesis theory that covers the disadvantages of CBT and PDT. For example, DCBT considers it necessary to establish a therapeutic alliance to achieve effective results from therapy sessions. Furthermore, unlike behavioural therapies, DCBT implies that “the tacit level of the information processing” affects daily life. On the other hand, it does not believe that this tacit process is psychologically dangerous, unlike psychodynamically oriented theories. In addition, DCBT also does not assume that insight per se is adequate for therapeutic change [26].

When examining the literature, the integration of CBT and various psychotherapy theories was observed, but there were not sufficient studies with dynamically oriented therapies and techniques. As Abbas and colleagues [18] state, the reason why dynamic methods and cognitive techniques have not been combined so far may be similar to prejudices towards PDT. For example, Larson [27] expressed this gap in the literature in a letter he wrote to a psychiatry journal's editor around 20 years ago as follows: “*Why no one has melded cognitive and dynamic theory in the grand fashion of our best but disparate cognitive and dynamic theorists. Call it ‘dynamic cognitive therapy.’ I can think of no better therapist than one capable of dynamic understanding and interpretation, who can use cognitive techniques to help patients put into action the fruits of their psychodynamic insights. This hybrid therapy no doubt occurs in offices around the globe, by accident or design. What is missing is academic sanction by formal attention and fleshing out of the details.”* He then encouraged the development of something new instead of using “*instruction manuals”* for performing therapies. Then, the journal’s editor received several responses on this issue, but no tangible work was conducted to fill the academic gap Larson expressed. Therefore, Larson [28] dropped the subject by expressing that he sets his hope of resolving this matter to the next generation of academics.

Turner [26] also emphasises that this integration of both psychoanalytic and cognitive behavioural therapy techniques would provide more effective treatment, and this new model will provide long-term gains that are more enduring. However, given the paucity of the literature, it is thought that it is important to work with a client diagnosed with SAD by using this approach. Therefore, this study aims to describe the almost forgotten DCBT approach step by step through a case report and to reveal the effectiveness of this approach.

## 2. Materials and Methods

### 2.1. Case Report

The client is a 21-year-old university student and is a member of a large family living in a rural area. His mother is his father’s second wife. While he was young, he had to study at a school far from his family. The client has a fragile and “inability to say no” personality trait. He expects to be appreciated by his father and other family members, but this expectation has not been met. His father is the person who does not understand him, and his mother is the person whose absence cannot be tolerated. He has been subjected to a lot of traumatising life events. He lost his brother five years ago, his nephew committed suicide two years ago and he lost his sister last year. He was directed to the psychotherapy sessions by a lecturer. The reasoning behind his referral was determined as the intense anxiety experienced by the client in the classroom. Furthermore, when this anxiety started to affect the client’s attendance in class and drove him to leave the university, he decided to seek professional help.

### 2.2. Therapeutic Process

After the client was verbally informed about the study, he signed the informed consent form. DCBT sessions consisting of nine sessions, two of which were anamnesis sessions, were held with him.

#### 2.2.1. Assessment of the Problem

- Measurement Tools

For the assessment of the patient, the following instruments were used.

1. Symptom Check List (SCL-90-R)

It is a valid and reliable measurement tool developed by Derogatis [29], adapted to more than 30 languages, and used in more than 2000 studies [30]. It is a 5-point Likert-type measuring instrument that takes values between 0 (none) and 4 (too much). It consists of 10 sub-dimensions: somatisation, obsessive-compulsive symptoms, interpersonal sensitivity, depression, anxiety, hostility, phobic anxiety, paranoid thinking, psychoticism, and additional scale (some other symptoms such as poor appetite are not covered by previous dimensions).

2. Beier Sentence Completion Test (B Form)

BSCT is a projective and semi-structured test developed by the American psychologist Delton C. Beier. There are two forms of BSCT which are A and B. BSCT-B form is applied to individuals aged 16 and over, and contains a total of 67 incomplete sentences [31]. Sentence completion tests are one of the most used methods for assessing personality [32]. Here, some incomplete sentences are presented to clients, and they are asked to complete the missing sentences. It is preferred that these missing parts are completed as quickly as possible to catch clients’ initial thoughts.

3. Beck Depression Inventory (BDI)

BDI is a measurement tool consisting of 21 items, including depressive symptoms such as pessimism, lack of satisfaction, feelings of failure and guilt, decreased appetite, fatigue, and social withdrawal [33]. It is a 4-point Likert-type self-assessment scale that is rated between 0 and 3. It is one of the most widely used measurement tools to determine the severity of depressive symptoms [34].

4. Anamnesis

One of the fundamentals of the dynamic-oriented methods is the necessity of history-taking from the client by asking many questions before the psychotherapy session begins [35,36,37,38]. The crucial instrument in the dynamic-oriented method is the anamnesis session, or sessions if necessary, so that the life story of the client would be revealed and an appropriate intervention technique would be developed [39]. However, knowing the hardships that the clients are going through and how they began is not enough. The history-taking questioning should be so meticulous that even the antenatal story and the possible future of clients are revealed together with their life stories [40].

#### 2.2.2. Therapeutic Goals and Intervention Strategies

After the anamnesis sessions and measurement tools were applied to the client, the first formulation for the psychotherapy sessions was created, and the process was started. Each therapeutic objective given below was revised after each session according to the client’s needs. After the end of each session, the therapist created a summary and evaluation report for that session, and then deciphered each session word for word. These transcripts and camera recordings were shared with the therapist’s supervisor before the next session, and the most appropriate intervention for the client was chosen. 

Therapeutic goals followed throughout the sessions were as follows:

- Establishing a therapeutic alliance required for therapy and motivating the client to attend psychotherapy sessions.
- Providing emotional catharsis and alleviating the client’s anxiety.
- Increasing the client’s insight into the similarities and differences between family relationships and other social relationships.
- Making the client aware of his tacit and overt cognitive processing and modifying his negative thoughts and cognitive distortion.
- Preparing the client to confront and cope with social phobic circumstances.

The following intervention strategies were used to achieve the goals given above.

- Applying some psychometric measurements.
- Examining the client’s entire life comprehensively, from pre-birth to now.
- Examining the family relationships of the client and understanding his attachment style.
- Explaining some of the basic CBT concepts and providing psycho-education to recognise cognitive distortions.
- Teaching and practising identifying automatic thoughts (A-B-C model).
- Applying imagination and systematic desensitisation techniques.
- Exposing the client to the situation that he is afraid to encounter.
- Modifying emotional and tacit cognitive schema.

## 3. Results

### 3.1. Sessions

#### 3.1.1. Before the Sessions

Before starting the psychotherapy sessions, the measurement tools mentioned above were applied to the client. Brief information was given about the psychotherapy process, and the first date of the clinical interview was clarified.

#### 3.1.2. First Two Sessions (Anamnesis)

*T: How would you describe the future that awaits you?*

*C: I never thought of the future!*

*T: Haven’t you thought about it lately?*

*C: I cannot think of it! I don’t have a future goal right now.*

In the first session, it was observed that the client was very timid, and his anxiety levels were high. He was not fully seated in his chair, his hands were constantly between his knees, and he was in a forward-bent body posture. This was also a position he often entered while describing his relationships with his father. His father has a total of 20 children from both wives. Losing his sister was the most painful event in his life. The client who has an anxious attachment style stated that he had a good relationship with his mother and that sometimes his mother spent time with him lying on his bed when he woke up in the mornings. One of the factors triggering his anxiety was seeing his mother die in his dreams. He thinks that he has not been receiving the attention he expected from his family, and he has been blamed too much by the family members, especially by his father.

To sum up, the anamnesis sessions were conducted in two sessions. In this process, a comprehensive story was taken from the pre-natal period of the client to now. More than a hundred questions which are about questioning family dynamics, self-design, sexual life, and the dreams he wished to realise were asked. The questions asked to the client made him feel that he was worthy of attention, and a therapeutic alliance was established. This step is significant because the first session’s essential role is for the therapist to check the client’s capacity to establish a healthy relationship [26].

#### 3.1.3. Third Session

*‘I heard the news of her death. I don’t want to believe it! As if there was no such thing… I slept and woke up… I don’t know… I am not even aware that I was sleeping. Such a thing cannot happen! She lives, among us! My eyes are always looking for her, but I cannot find it…’*

The anamnesis process was summarised to the client, and it was stated that the next sessions are psychotherapy sessions, therefore he will not be asked many questions unless necessary. The orienting started with the metaphor of “a blind man and a suburban train”. At this point, this story explained what the roles of the client and the therapist should be, and his insight was tested by receiving feedback from him. This metaphor is about the journey of a blind old man and a young woman on a suburban train. The story is summarised as follows [37,39]: The blind man offers to make their trip more enjoyable for the young woman. The rule of the game is simple. Each time the train stops to pick up passengers, the young woman will describe everything around the train to him, and he will tell her where she is. The game starts. The young woman starts to explain everything she sees, and the old blind man guesses correctly. Then, when they reach a long dark tunnel, the young woman stops to describe it. After passing it, she begins to describe everything again, but this time the old man does not know, and the young woman cries “No! You could not know this time!” “But how come?” asks the old man and adds “Did we go through a long dark tunnel?” The young woman says “Yes…!” amazedly. Then, “You would tell me everything you saw. We had agreed like this. If you do not tell me what you saw, of course, I would not know!” says the old blind man, and the story ends like that.

After the story, it is asked to the client:

*T: So, who is the young passenger in this story?*

*C: Umm, I guess I am (laughs).*

*T: Old man?*

*C: That is you too.*

*T: Because?*

*C: Because… I am going to tell you what I see, and you will make me realise the events that I cannot see or understand.*

*T: Great! Let’s start. The train is moving… Choo, Choo, Choo…*

After this introduction, it was observed that the client expressed his feelings more sincerely and without manipulating himself. In this session, the death of his sister was discussed. He stated that his sister had joined a terrorist organisation and was later killed. He was allowed to talk about his memories with his sister and go through the mourning process. Then, the reasons underlying the sentence he completed as “Men are more fortunate than women!” was worked on with him. This sentence was extracted from BSCT which is one of the incomplete sentences and starts with “Men……”. It was observed that he felt intense anger towards his father and blamed his father for the death of his sister. However, the session ended by expressing that he respected his father because his superego was dominant. The statements of the client regarding the death of his sister were as follows:

*“If she was a boy, she could go to school. My father did not allow her to get an education just because she was a girl. However, if she had studied, she would have been a very successful and educated person and would not have had to go to the mountains. It would not end like this either.”*

#### 3.1.4. Fourth Session

*“I used to be comfortable in front of everyone. Now, why did I become like this? I don’t understand. I don’t want to stay behind in my education life. I want to be a good role model for my siblings.”*

In this session, the client talked about the impact of his brother’s death on him. He described how his brother died traumatically while he was working in the field, by being crushed under a rock rolling from a mountain. In this emotion-focused session, traumatic events thought to cause anxiety were discussed, and catharsis was ensured. Moreover, in this session, it was observed that the client experienced the feeling of “industry versus inferiority” and made these emotions more manageable by using a “projection” defence mechanism. For example, he claimed that the reason for failing to pass the university entrance exam in the first years was “the evil eye of the peasants” and continued: “*I could not pass, because before I took the exam, the peasants said that I would definitely pass the exam and place to the law department at the first entry. That’s why I failed. Due to their evil eye…*”

#### 3.1.5. Fifth Session

*“That day… I wish I didn’t go to that lecture!”*

In this session, the dynamics that caused the client to not want to attend lectures and leave the university were discussed. He stated that the lecturer had asked him to read a text in front of the classroom and that he was very excited and fainted during this reading. When asked “Would you go back to that moment?, What is going on?”, after waiting for a while, he summarised his situation as follows: “*I say to myself I will be disgraced, and in the end, I am disgraced by falling on the ground. Since that incident, my friends make fun of me every time they see me. I cannot attend lectures anymore because almost every lecturer wants us to make presentations. I cannot speak in front of a huge class!*”

Intensive cognitive therapy is initiated in the middle stages of treatment. Patients are trained to understand and track their tacit and overt cognitions [26]. Therefore, this session aims to use the findings regarding the formulation and conceptualisation established in the first two sessions in a way to increase the insight of the client. Accordingly, a link was established between his need to be appreciated within the family and the need to be appreciated in his social environment. Finally, a brief psycho-education was provided about “irrational beliefs” by drawing attention to the cognitive distortions underlying his anxiety, and the session was ended.

#### 3.1.6. Sixth Session

*“Today, I visited one of my lecturers in his office. My friends also were with me. He said it seems I have changed a lot, and my conversations had become quite confident.”*

The “catching automatic thoughts” technique used in cognitive behavioural therapy was attempted to be reinforced based on the A-B-C model, and examples were taken from the client’s life. Additionally, his self-awareness and therapy process were discussed in this session. The client expressed that he was aware of the change in himself and emphasised that he could see this more clearly when one of his lecturers stated this change.

#### 3.1.7. Seventh Session

*“I feel comfortable here. Before I came here, I had lost my self-confidence, I had lost everything. But now I’m getting better. I can see that…”*

The late middle stage of therapy is concentrated on developing the self-management capabilities of clients. During this process, behavioural intervention strategies such as cognitive skills training, problem-solving instruction, and systematic desensitisation are applied. These strategies are not seen as simply a means of modifying behaviour. Rather, DCBT sees them as effective methods for altering emotional and tacit cognitive schemes [26].

In this session, the client’s anxiety was studied more intensely. Various CBT techniques have been used, such as imagination, assuming the worst will happen, and examining the evidence (evidence that supports and does not support negative thoughts). When the client said that he had to make a presentation in a lecture in the coming weeks, his fears regarding this issue were discussed, and the client was given imaginary rehearsals. As the duration and repetition of the imagery increased, the intensity of his anxiety began to decrease. He was asked to do these imaginary rehearsals for a week and imagine going to an empty classroom at least twice and giving a presentation.

#### 3.1.8. Eighth Session

*T: You say that you have made a lot of progress in this process, learned the techniques here, and felt more comfortable than before.*

*C: Yes!*

*T: So how about bursting into a classroom now?*

*C: How so?*

*T: Now we will get up, go faculty and enter a class, and you will explain, for example, what the A-B-C model is to them!*

*C: You are joking, I guess?*

Considering the progress of the client, the “Exposure” technique was used in this session. The client was asked to explain the “catching automatic thoughts” technique, which he had learned in the process in a lecture he did not know at all. In the class, the therapist initiated the lesson in partnership with the client. The purpose of the therapist to do this was to confront the client with his fear by making him feel that he was not alone.

He expressed his feelings triumphantly, after this exposure as follows:

*“Actually, it was not that hard, I was very excited at first, but now I’m so comfortable that I cannot tell. I thought I was a little stuck but still fine, I guess. How crowded was the class? How did you set it up? At first, it felt like it was planned when the positive reviews came. But when I heard the next criticisms and also saw the students coming late to the class, I thought that these weren’t plans. Here I am very comfortable right now.”*

#### 3.1.9. Ninth Session (Termination)

*“After the session, we reserved some seats in a cinema with friends for a comedy movie. It will start at 13.30.”*

The last phase of treatment focuses on problems of closure and prevention of regression. The termination process is seen as the time of consolidation of the progress and improvements achieved in counselling. The closure process is perceived to be a period of consolidation of success and change in therapy [26]. In this last session, the client’s achievements in the therapy sessions and his feelings about ending the sessions were reviewed. It was observed that the client was comfortable and able to tolerate separation anxiety regarding the termination.

*“I am very comfortable right now, I have reached a level where I can make presentations in any lecture, and I started to socialise with my friends again. You have said before that the termination of these sessions means that I am getting better, and it does not mean that we will not meet again. Also, I already know where I can find you (Laughs).”*

Finally, his victory in the previous session was discussed, and the process was completed with positive emotions.

### 3.2. Quantitative (Descriptive) Results

**Figure 1:**
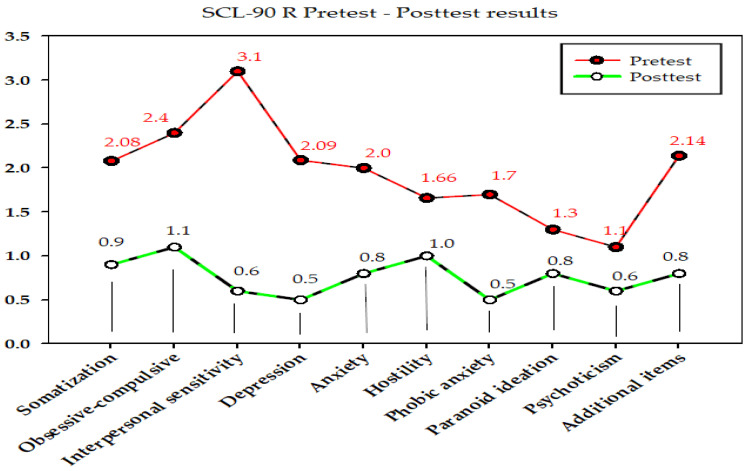
Comparisons of the SCL-90-R pretest-posttest results.

**Figure 2:**
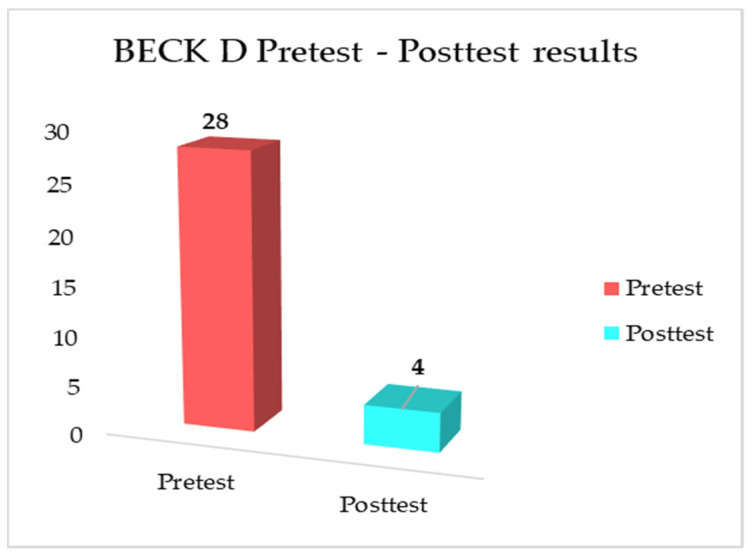
Comparisons of the BDI pretest-posttest results.

Comparisons of the SCL-90-R and BDI pretest-posttest results of the client are shown in Figure 1 and Figure 2 below.

When comparing the pre-test and post-test results (Figure 1 and Figure 2), it was observed that the level of depression (BDI pre-test vs. post-test: 28 vs. 4; SCL-90-R depression subscale: 2.09 vs. 0.5) and psychological symptoms of the client (SCL-90-R subscale: pre-test vs. post-test; somatisation: 2.08 vs. 0.9; obsessive-compulsive: 2.4 vs. 1.1; interpersonal sensitivity: 3.1 vs. 0.6; anxiety: 2.0 vs. 0.8; hostility: 1.66 vs. 1.0; phobic anxiety: 1.7 vs. 0.5; paranoid ideation: 1.3 vs. 0.8; psychoticism: 1.1 vs. 0.6; additional items: 2.14 vs. 0.8) decreased, and the positive expressions in the Beier sentence completion test increased after the DCBT sessions. It has been seen that DCBT is efficient in solving the problem of the client with SAD. It increased the client’s insight and reduced his distorted thoughts; thus, he could re-establish social harmony. Realising new learnings, he has also begun to be more sociable in living new experiences.

## 4. Discussion

This paper proposes the integration of cognitive behavioural and psychodynamic theory and technique in treatment and demonstrates such integration with a nine-session therapy of a young man suffering from a social anxiety disorder, applying dynamic cognitive behavioural psychotherapy (DBCT).

In this paper, therapeutic objectives and psychotherapeutic strategies were clearly established, and a series of instruments were used as tools to assess the problems before and after the interventions, showing improvement. The client has been through important and traumatic problems, and the treatment is based on accurate facilitative interventions so that important issues are seen in a few sessions.

It is promising to see how dynamic concepts can be integrated into the framework of cognitive therapies, as its content is remarkably interesting, and how psychoanalytic concepts integrate with other ways of working with patients. On the other hand, it should be emphasised that a single case offers little support for a general thesis. Furthermore, there is no follow-up in the interval after treatment.

Finally, it is believed that this paper is going to be an effective starting point for closing the gap mentioned because it aims both to reveal the nature of a counselling/therapy process and to include scientific discussions in order to discover common ground between theorists and clinicians. In this sense, with the mission of this paper, it may be possible to re-open an unanswered topic directed to the editor of the American Journal of Psychiatry nearly two decades ago, among both theorists and clinicians.

## 5. Conclusions

In this study, dynamically oriented cognitive behavioural therapy sessions were conducted to solve the problem of a client with SAD. Despite the limitations mentioned above, DCBT seems to be effective in the treatment of social anxiety disorders. Other researchers may conduct new studies to test the effectiveness of DCBT in other different mental health issues. The absence of monitoring in this study is a limitation. Therefore, researchers who will carry out similar research can conduct monitoring sessions and experimental studies to test the effectiveness and permanence of DCBT.

## Data Availability

The author has the video recordings of the therapy sessions, some transcriptions, and summaries of the content of each session as data and he can only share the abstract of each session upon reasonable request.

## References

[1] Jefferies P., Ungar M. (2020). Social anxiety in young people: A prevalence study in seven countries. PLoS ONE.

[2] Kessler R.C., Berglund P., Demler O., Jin R., Merikangas K.R., Walters E.E. (2005). Lifetime prevalence and age-of-onset distributions of DSM-IV disorders in the National Comorbidity Survey Replication. Arch. Gen. Psychiatry.

[3] Koyuncu A., İnce E., Ertekin E., Tükel R. (2019). Comorbidity in social anxiety disorder: Diagnostic and therapeutic challenges. Drugs Context.

[4] Alfonso S.V., Alfonso L.A., Llabre M.M., Fernandez M.I. (2015). Project stride: An equine-assisted intervention to reduce symptoms of social anxiety in young women. Explore.

[5] Bandelow B., Michaelis S., Wedekind D. (2017). Treatment of anxiety disorders. Dialogues Clin. Neurosci..

[6] Butler R.M., O’Day E.B., Swee M.B., Horenstein A., Heimberg R.G. (2020). Cognitive behavioral therapy for social anxiety disorder: Predictors of treatment outcome in a quasi-naturalistic setting. Behav. Ther..

[7] Rahmani F., Abbass A., Hemmati A., Mirghaed S.R., Ghaffari N. (2020). The Efficacy of Intensive Short-Term Dynamic Psychotherapy for Social Anxiety Disorder: Randomized Trial and Substudy of Emphasizing Feeling Versus Defense Work. J. Nerv. Ment. Dis..

[8] APA APA Dictionary of Psychology: Cognitive Behavior Therapy (CBT). https://dictionary.apa.org/cognitive-behavior-therapy.

[9] APA APA Dictionary of Psychology: Psychodynamic Psychotherapy. https://dictionary.apa.org/psychodynamic-psychotherapy.

[10] Lindegaard T., Hesslow T., Nilsson M., Johansson R., Carlbring P., Lilliengren P., Andersson G. (2020). Internet-based psychodynamic therapy vs cognitive behavioural therapy for social anxiety disorder: A preference study. Internet Interv..

[11] Hayes S.C., Hofmann S.G. (2018). Process-Based CBT: The Science and Core Clinical Competencies of Cognitive Behavioral Therapy.

[12] Fonagy P. (2015). The effectiveness of psychodynamic psychotherapies: An update. World Psychiatry.

[13] Paris J. (2005). Fall of an Icon: Psychoanalysis and Academic Psychiatry.

[14] Paris J. (2017). Is psychoanalysis still relevant to psychiatry?. Can. J. Psychiatry.

[15] Wolpert L., Fonagy P. (2009). There is no place for the psychoanalytic case report in the British Journal of Psychiatry. Br. J. Psychiatry.

[16] Steinert C., Munder T., Rabung S., Hoyer J., Leichsenring F. (2017). Psychodynamic therapy: As efficacious as other empirically supported treatments? A meta-analysis testing equivalence of outcomes. Am. J. Psychiatry.

[17] Blackmore C., Beecroft C., Parry G., Booth A., Tantam D., Chambers E., Simpson E., Roberts E., Saxon D. (2009). A Systematic Review of the Efficacy and Clinical Effectiveness of Group Analysis and Analytic/Dynamic Group Psychotherapy.

[18] Abbass A., Luyten P., Steinert C., Leichsenring F. (2017). Bias toward psychodynamic therapy: Framing the problem and working toward a solution. J. Psychiatr. Pract..

[19] Johnsen T.J., Friborg O. (2015). The effects of cognitive behavioral therapy as an anti-depressive treatment is falling: A meta-analysis. Psychol. Bull..

[20] Cristea I.A., Stefan S., Karyotaki E., David D., Hollon S.D., Cuijpers P. (2017). The effects of cognitive behavioral therapy are not systematically falling: A revision of Johnsen and Friborg (2015). Psychol. Bull..

[21] Speake J., Simpson J. (2008). A Dictionary of Proverbs.

[22] Feixas G., Botella L. (2004). Psychotherapy integration: Reflections and contributions from a constructivist epistemology. J. Psychother. Integr..

[23] Giovazolias T. (2004). The therapeutic relationship in cognitive-behavioural therapy. Couns. Psychol. Rev.-Br. Psychol. Soc..

[24] Woody S.R., Adessky R.S. (2002). Therapeutic alliance, group cohesion, and homework compliance during cognitive-behavioral group treatment of social phobia. Behav. Ther..

[25] Pilecki B., Thoma N., McKay D. (2015). Cognitive behavioral and psychodynamic therapies: Points of intersection and divergence. Psychodyn. Psychiatry.

[26] Turner R.M., Giles T.R. (1993). Dynamic cognitive-behavior therapy. Handbook of Effective Psychotherapy.

[27] Larson E.W. (1992). Dynamic cognitive therapy anyone?. Am. J. Psychiatry.

[28] Larson E.W. (1993). On dynamic cognitive therapy. Am. J. Psychiatry.

[29] Derogatis L.R. (1977). The SCL-90 Manual I: Scoring, Administration and Procedures for the SCL-90.

[30] Derogatis L.R., Unger R. (2010). Symptom checklist-90-revised. Corsini Encycl. Psychol..

[31] Akkoyun F. (2014). Projective Tests.

[32] Holaday M., Smith D.A., Sherry A. (2000). Sentence completion tests: A review of the literature and results of a survey of members of the society for personality assessment. J. Personal. Assess..

[33] Beck A.T., Ward C.H., Mendelson M., Mock J., Erbaugh J. (1961). An inventory for measuring depression. Arch. Gen. Psychiatry.

[34] Toledano-Toledano F., Contreras-Valdez J.A. (2018). Validity and reliability of the Beck depression inventory II (BDI-II) in family caregivers of children with chronic diseases. PLoS ONE.

[35] Bellak L. (1992). Handbook of Intensive Brief and Emergency Psychotherapy.

[36] Kaya M.S., Yıldırım T. (2021). Gelişimsel krize müdahalede kısa-yoğun-acil psikoterapinin kullanımı: Bir olgu sunumu [Using brief-emergency and intensive psychotherapy in intervention to the developmental crisis: A case study]. AYNA Klin. Psikol. Derg..

[37] Kaya M.S., Yıldırım T. (2022). Dinamik yönelimli yaklaşımlarda anamnez oturumundan psikoterapi oturumuna geçiş süreci: Aday psikoterapistler için bir kılavuz [Transition process from anamnesis session to psychotherapy in dynamically oriented approaches: A guide for candidate psychotherapists]. AYNA Klin. Psikol. Derg..

[38] Yıldırım T. (2013). A process of short-term dynamics oriented and individual counseling: A case of stuttering. Int. J. Psychol. Couns..

[39] Doğan Y.B. (2015). Kısa Acil Psikoterapi [Brief Emergency Psychotherapy (BEP)].

[40] Morrison J. (2008). The First Interview: A Guide for Clinicians.

